# Supplementary material for: The FAM53C/DYRK1A axis regulates the G1/S transition of the cell cycle
Source: eLife. 2026 Apr 30;14:RP109708. doi: 10.7554/eLife.109708 (PMC13132546; doi:10.7554/eLife.109708)

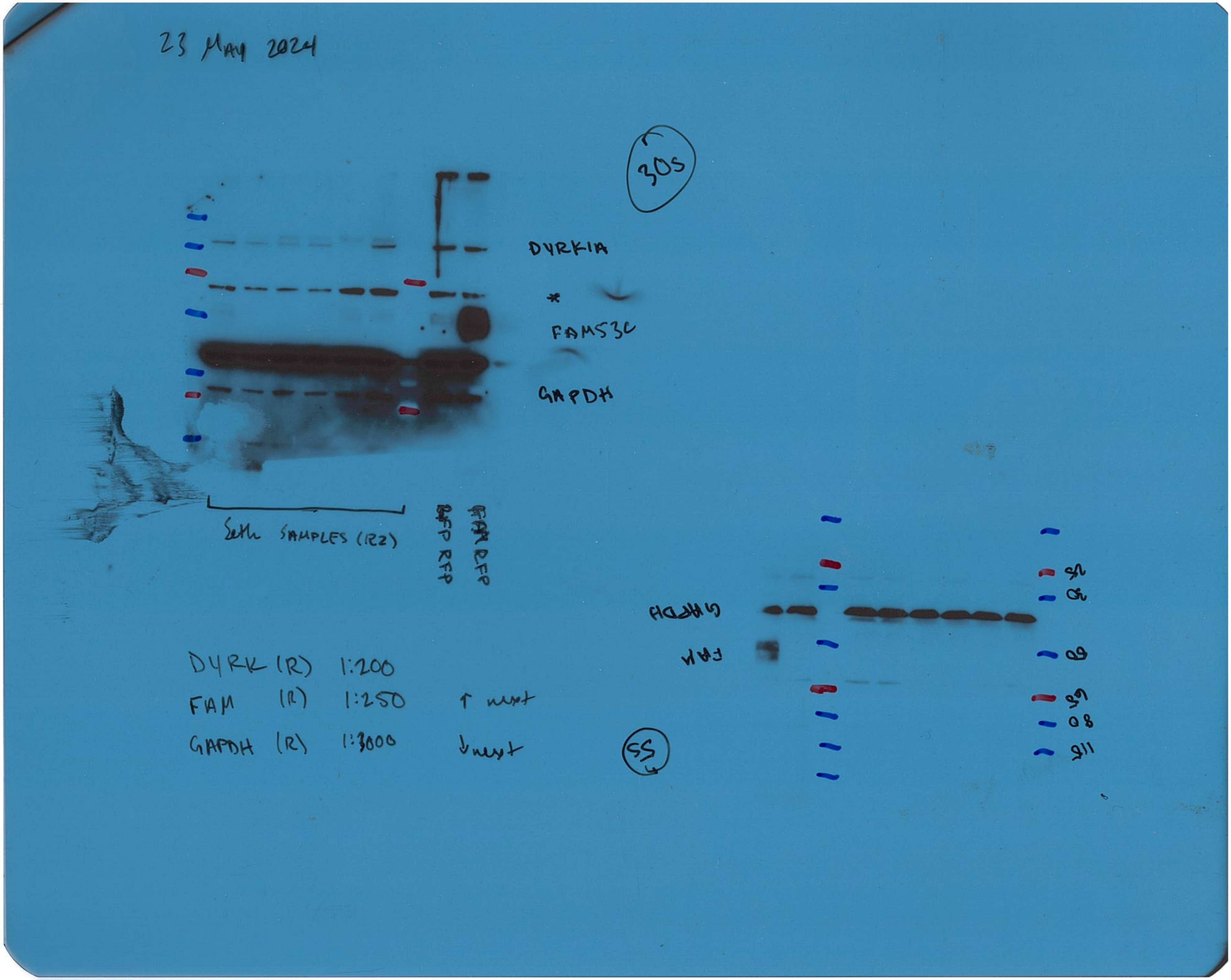
23 May 2024
30s
DYRK1A
*
FAM53C
GAPDH
Seth SAMPLES (R2)
RFP RFP
FAM RFP
DYRK (R) 1:200
FAM (R) 1:250 ↑ next
GAPDH (R) 1:3000 ↓ next
5s

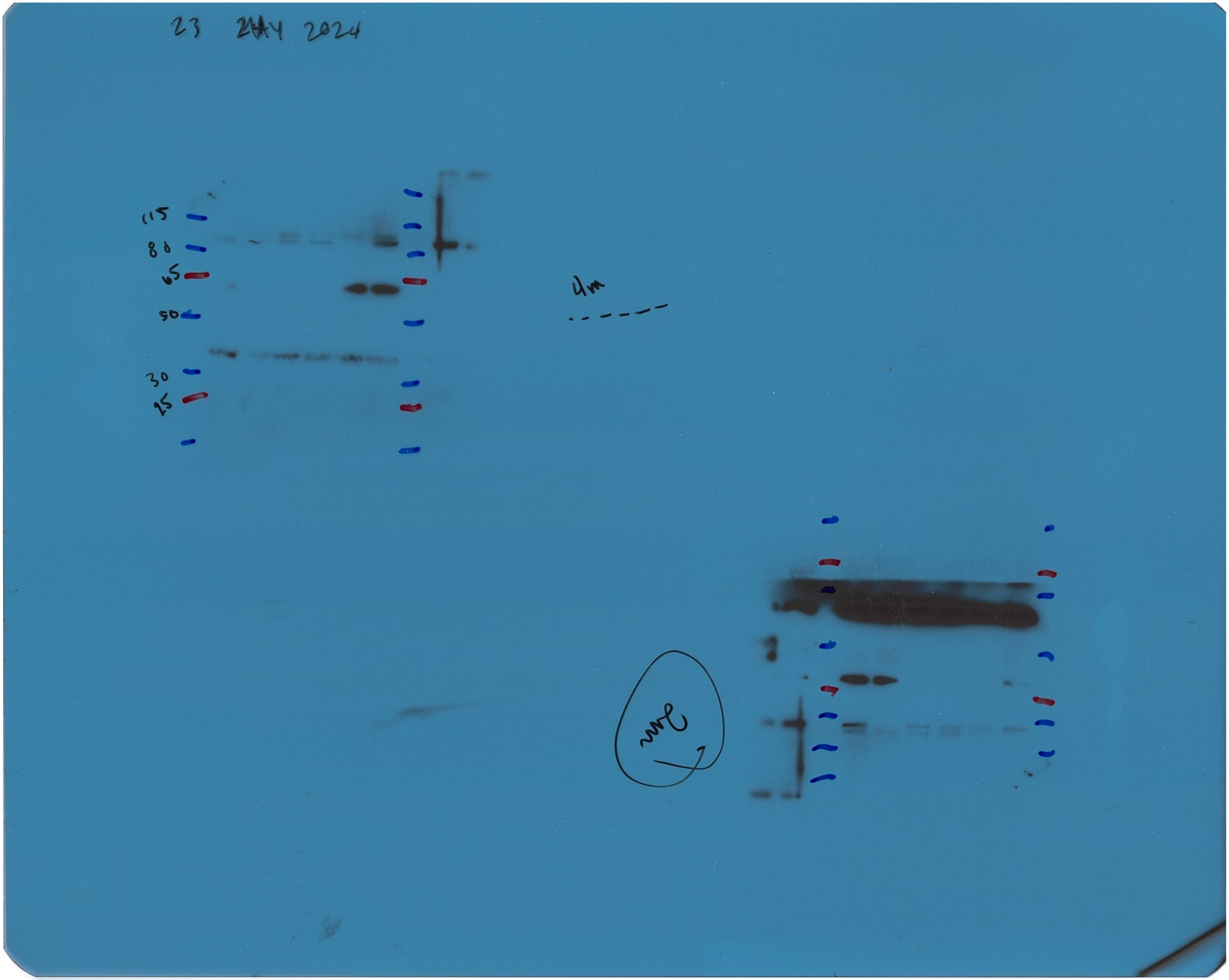

23 MAY 2024
115
80
65
50
30
25
4m
2m

23 May 2024

1:3000 GAPDH (R)

1:1000 HA (R)

1:1000 FLAG (M)

↳ 2°@ 1:5000

55

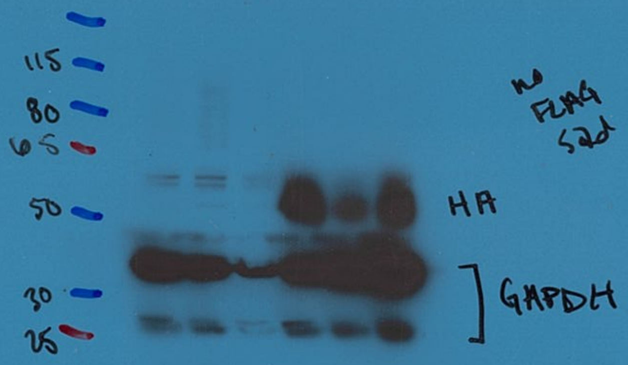

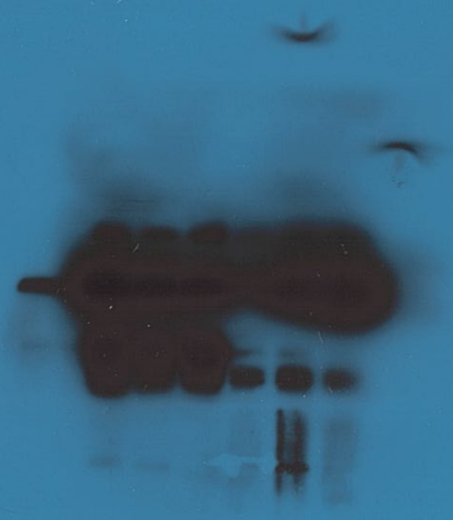

Supplement: Figure 1—source data 1. [file elife-109708-fig1-data1.zip › Figure 1 - Source data 1/Xerox Scan_05282024131028.pdf]
