# Supplementary figures and images for: The FAM53C/DYRK1A axis regulates the G1/S transition of the cell cycle
Source: eLife. 2026 Apr 30;14:RP109708. doi: 10.7554/eLife.109708 (PMC13132546; doi:10.7554/eLife.109708)

8 May 2024

1. siCtrl 24h  
 2. siFAM53C 24h  
 3. siCtrl 48h  
 4. siFAM53C 48h

\* Non specific?

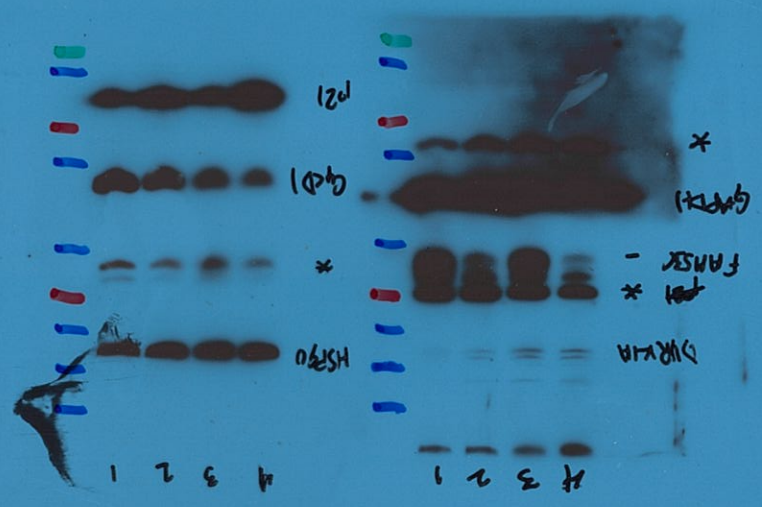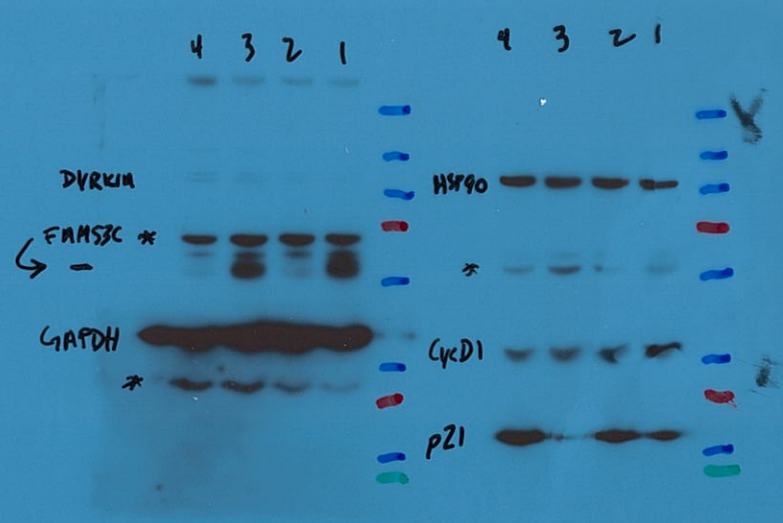

Supplement: Figure 1—source data 1. [file elife-109708-fig1-data1.zip › Figure 1 - Source data 1/Xerox Scan_05082024153659.pdf]

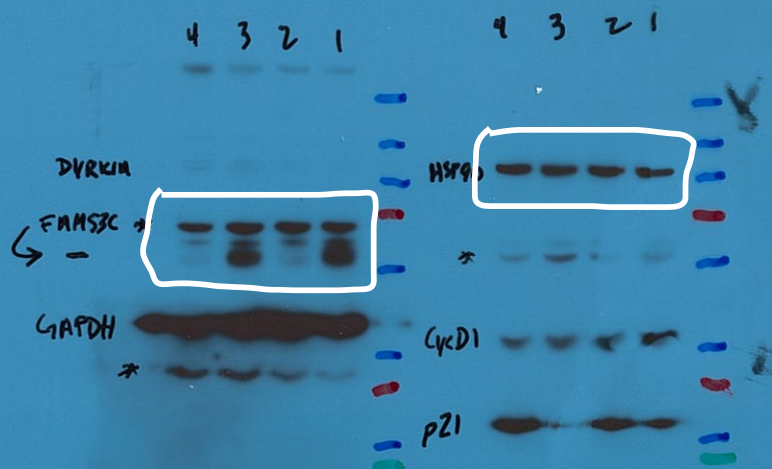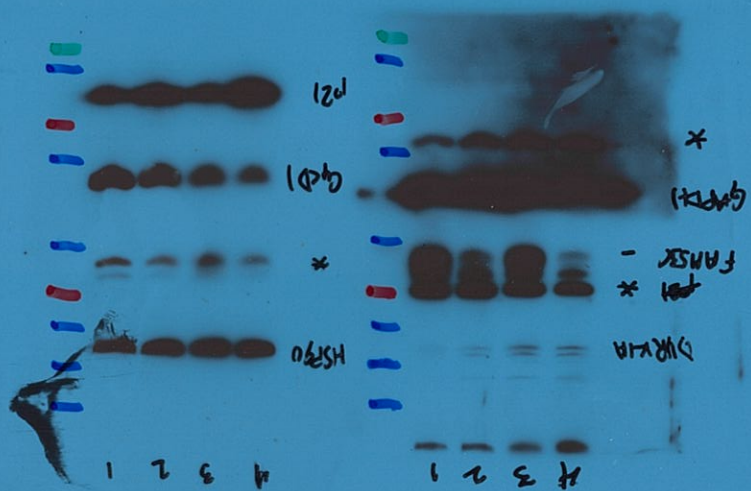

\* Non specific?

1. siCtrl 24h  
 2. siFAM53C 24h  
 3. siCtrl 48h  
 4. siFAM53C 48h

Supplement: Figure 1—source data 2. [file elife-109708-fig1-data2.zip › Figure 1 - Source data 2/Xerox Scan_05082024153659 with marks.pdf]

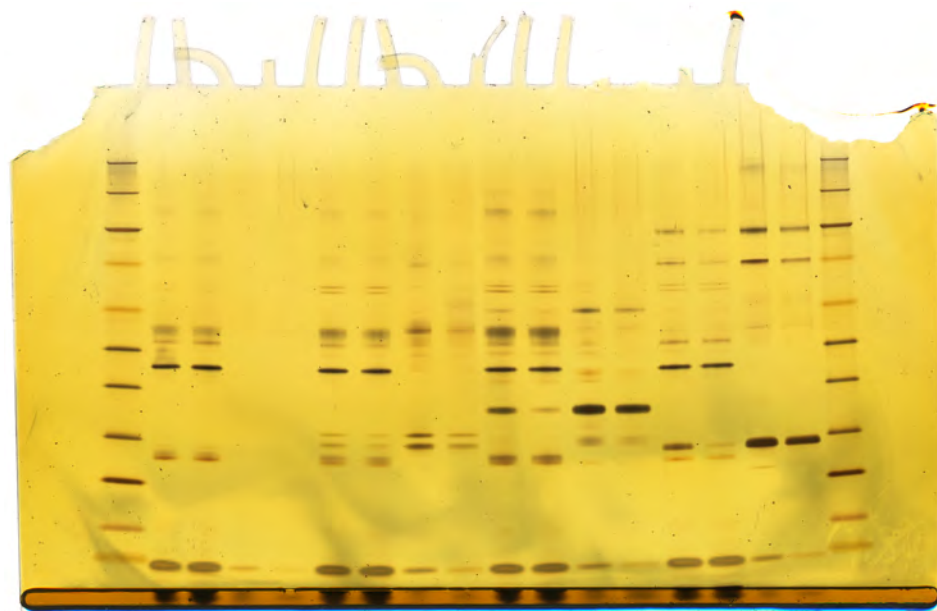

Supplement: Figure 2—figure supplement 1—source data 1. [file elife-109708-fig2-figsupp1-data1.zip › Figure 2 - figure supplement 1 - Source data 1/Figure2-supplement1A raw blots without labelling.pdf]

# Figure 2-figure supplement 1

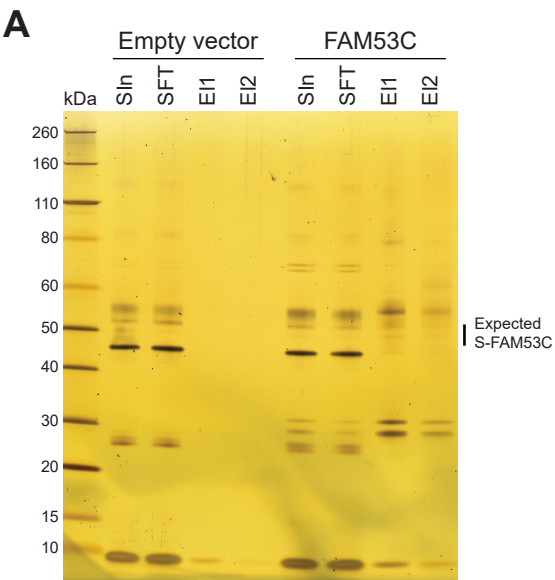

Supplement: Figure 2—figure supplement 1—source data 2. [file elife-109708-fig2-figsupp1-data2.zip › Figure 2 - figure supplement 1 - Source data 2/Figure2-supplement1A blots with labelling.pdf]

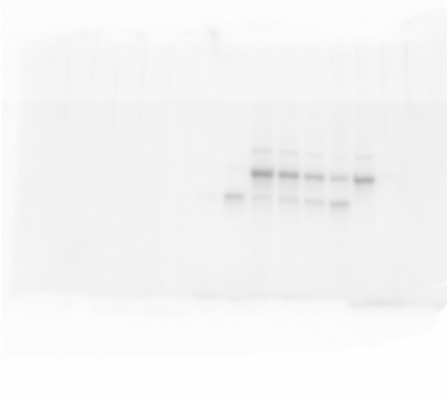

Supplement: Figure 3—source data 1. [file elife-109708-fig3-data1.zip › Figure 3 - source data 1/Kinase Assay CCND1.jpeg]

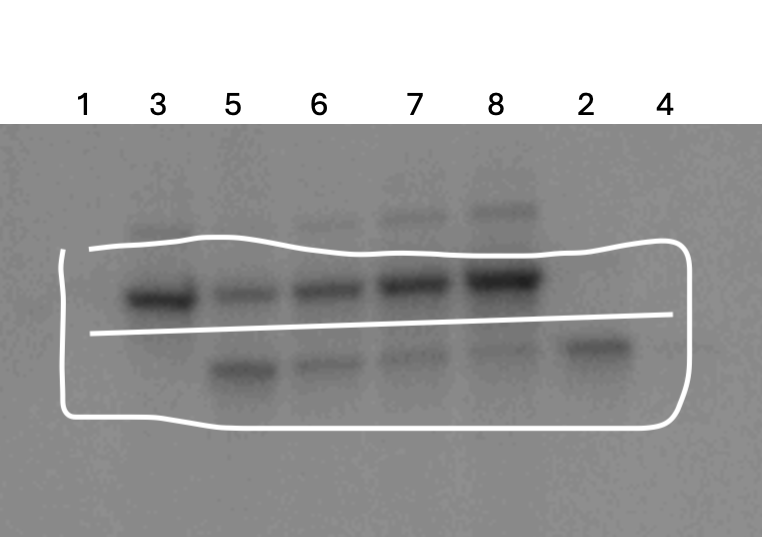

Supplement: Figure 3—source data 2. [file elife-109708-fig3-data2.zip › Figure 3 - source data 2/Kinase Assay CCND1 with marks.png]

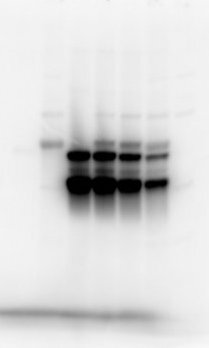

Supplement: Figure 3—figure supplement 1—source data 1. [file elife-109708-fig3-figsupp1-data1.zip › Figure 3 - figure supplement 1 - Source data 1/Kinase Assay Lin52.jpg]

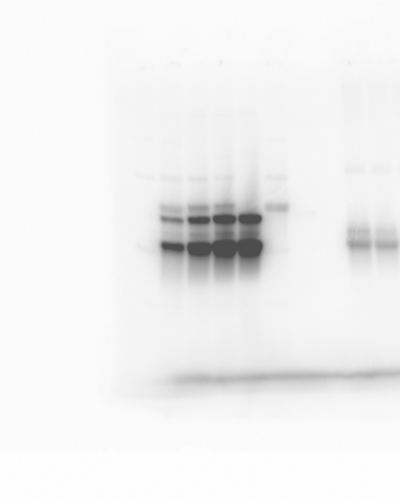

Supplement: Figure 3—figure supplement 1—source data 1. [file elife-109708-fig3-figsupp1-data1.zip › Figure 3 - figure supplement 1 - Source data 1/Kinase Assay Lin52.gel]

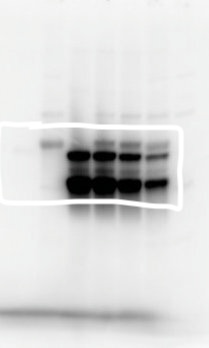

Supplement: Figure 3—figure supplement 1—source data 2. [file elife-109708-fig3-figsupp1-data2.zip › Figure 3 - figure supplement 1 -Source data 2/Kinase Assay Lin52 with marks.jpg]

5m

2m

5m

 $2m$ 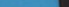



501

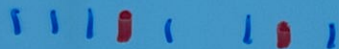

Supplement: Figure 4—figure supplement 1—source data 1. [file elife-109708-fig4-figsupp1-data1.zip › Figure 4 - figure supplement 1 - Source data 1/Xerox Scan_08052025135450.pdf]

Figure 5E

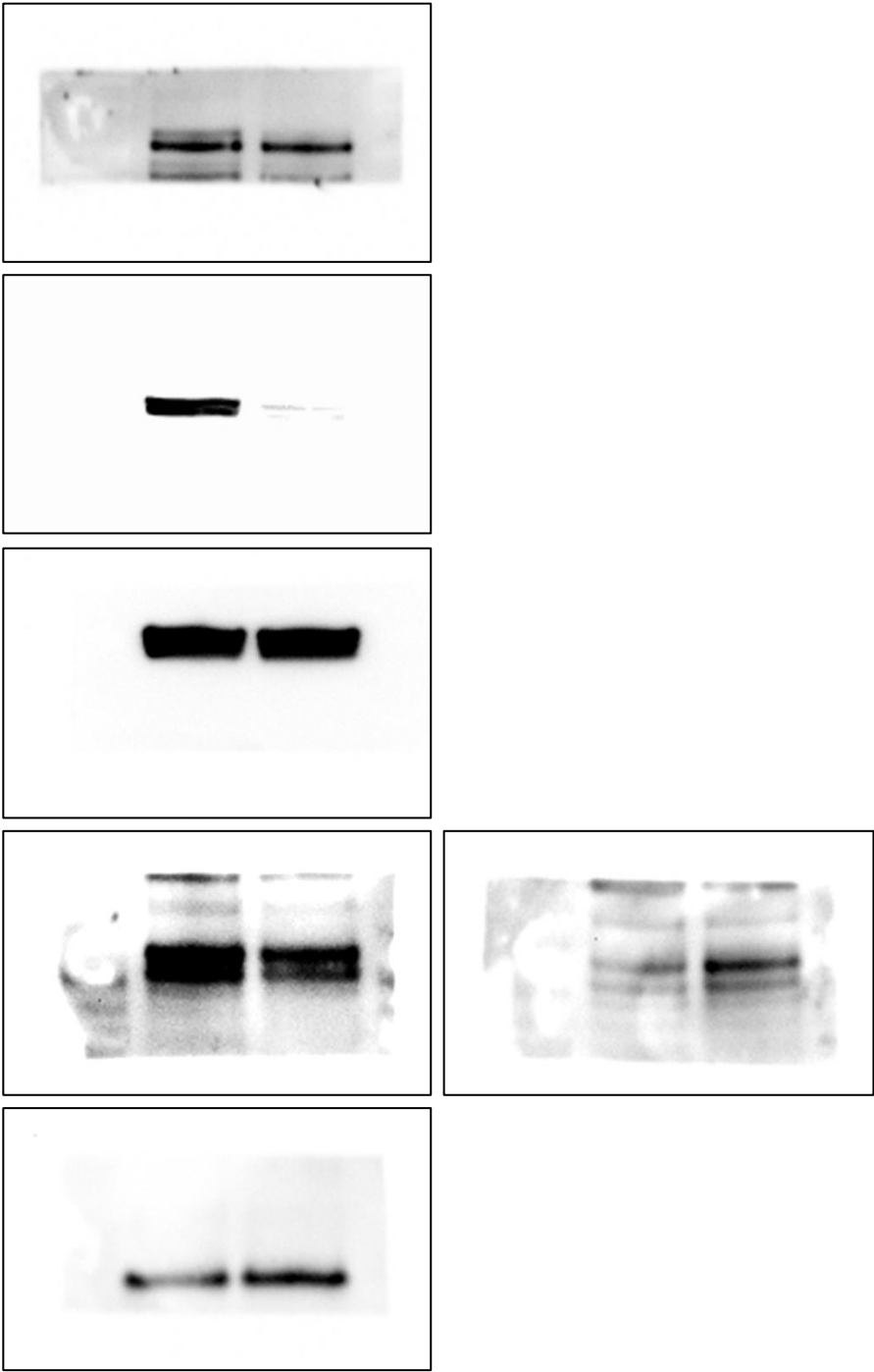

Supplement: Figure 5—source data 1. [file elife-109708-fig5-data1.zip › Figure 5 - source data 1/Figure5E raw blots without labeling.pdf]

Figure 5E

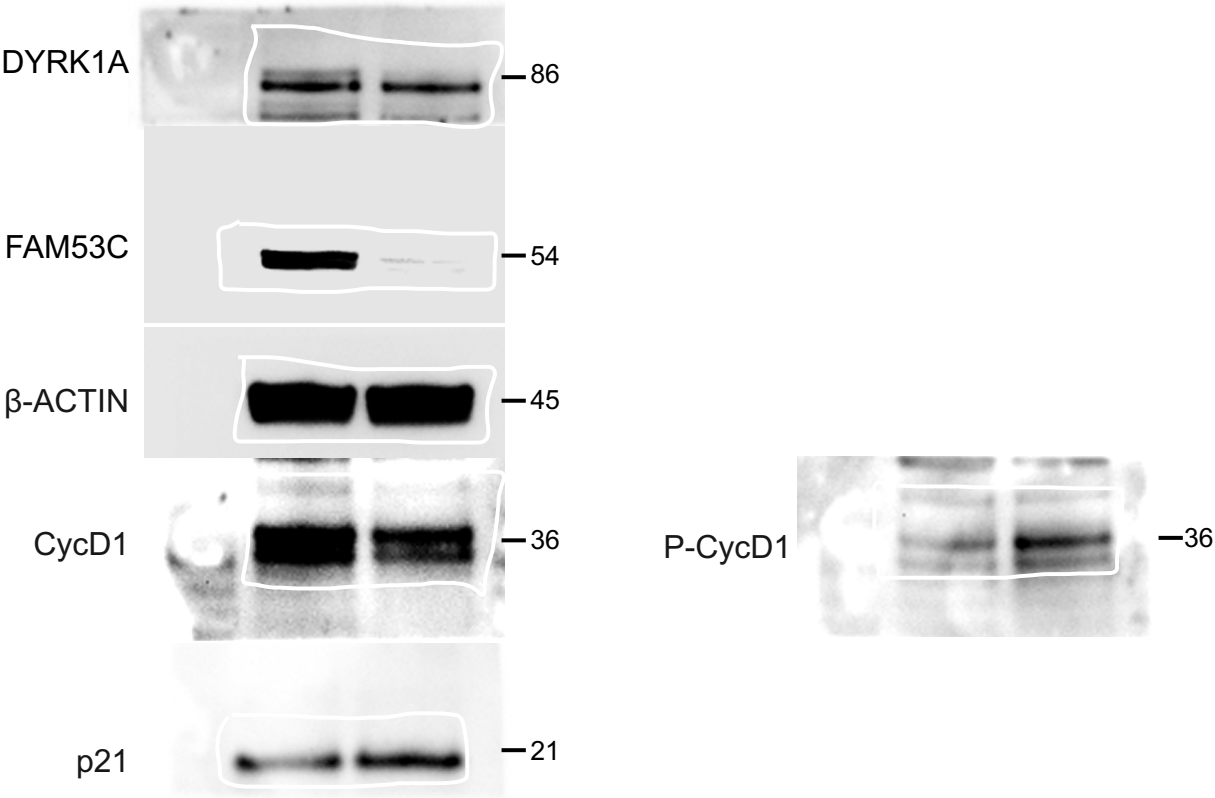

Supplement: Figure 5—source data 2. [file elife-109708-fig5-data2.zip › Figure 5 - source data 2/Figure5E raw blots with marks.pdf]
