# Supplementary material for: The FAM53C/DYRK1A axis regulates the G1/S transition of the cell cycle
Source: eLife. 2026 Apr 30;14:RP109708. doi: 10.7554/eLife.109708 (PMC13132546; doi:10.7554/eLife.109708)
Supplement: Figure 1—source data 2. [file elife-109708-fig1-data2.zip › Figure 1 - Source data 2/Xerox Scan_05282024131028 with marks.pdf]

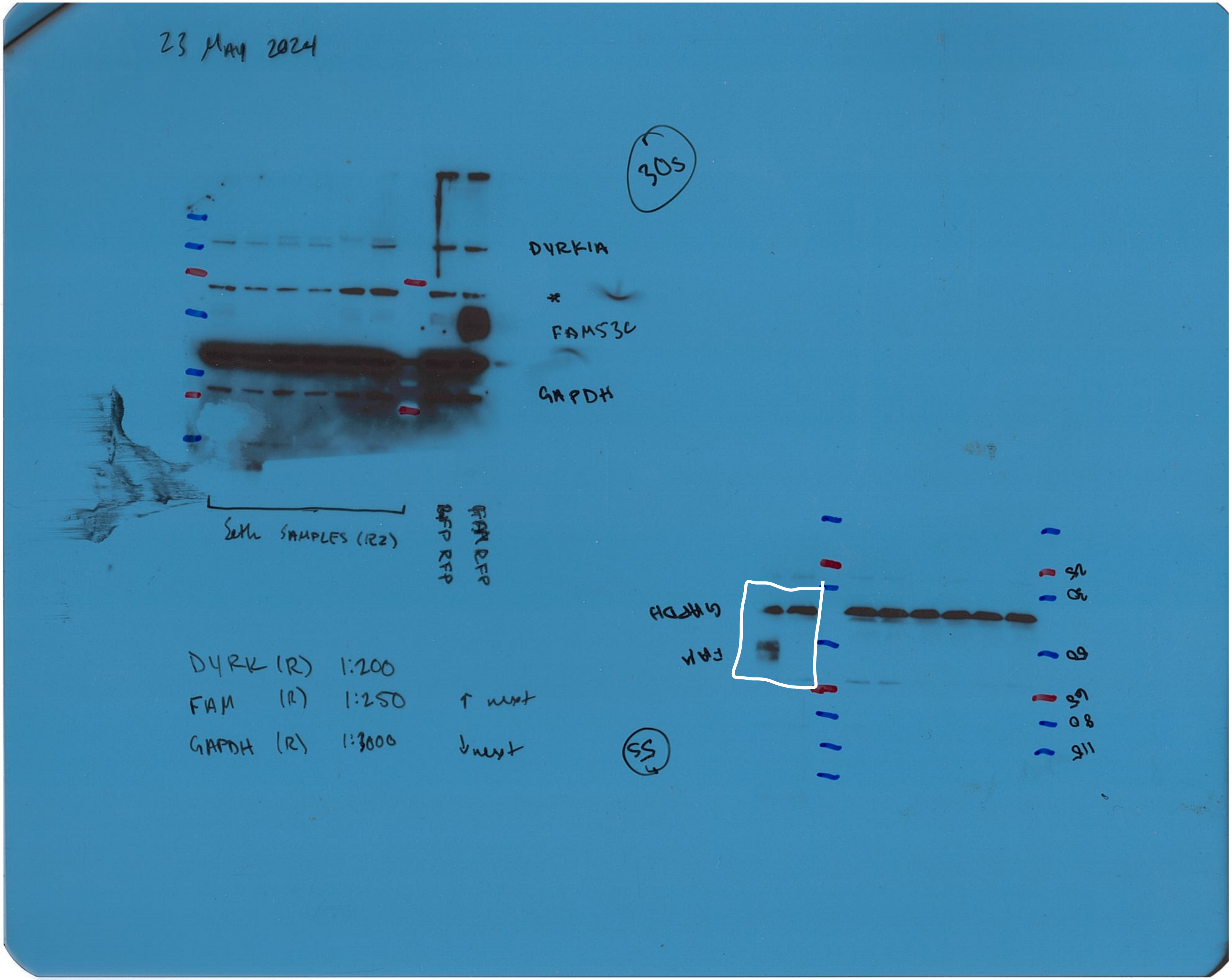
23 May 2024
30s
DYRK1A
*
FAM53C
GAPDH
Seth SAMPLES (R2)
RFP RFP
FAM RFP
DYRK (R) 1:200
FAM (R) 1:250 ↑ next
GAPDH (R) 1:3000 ↓ next
5s

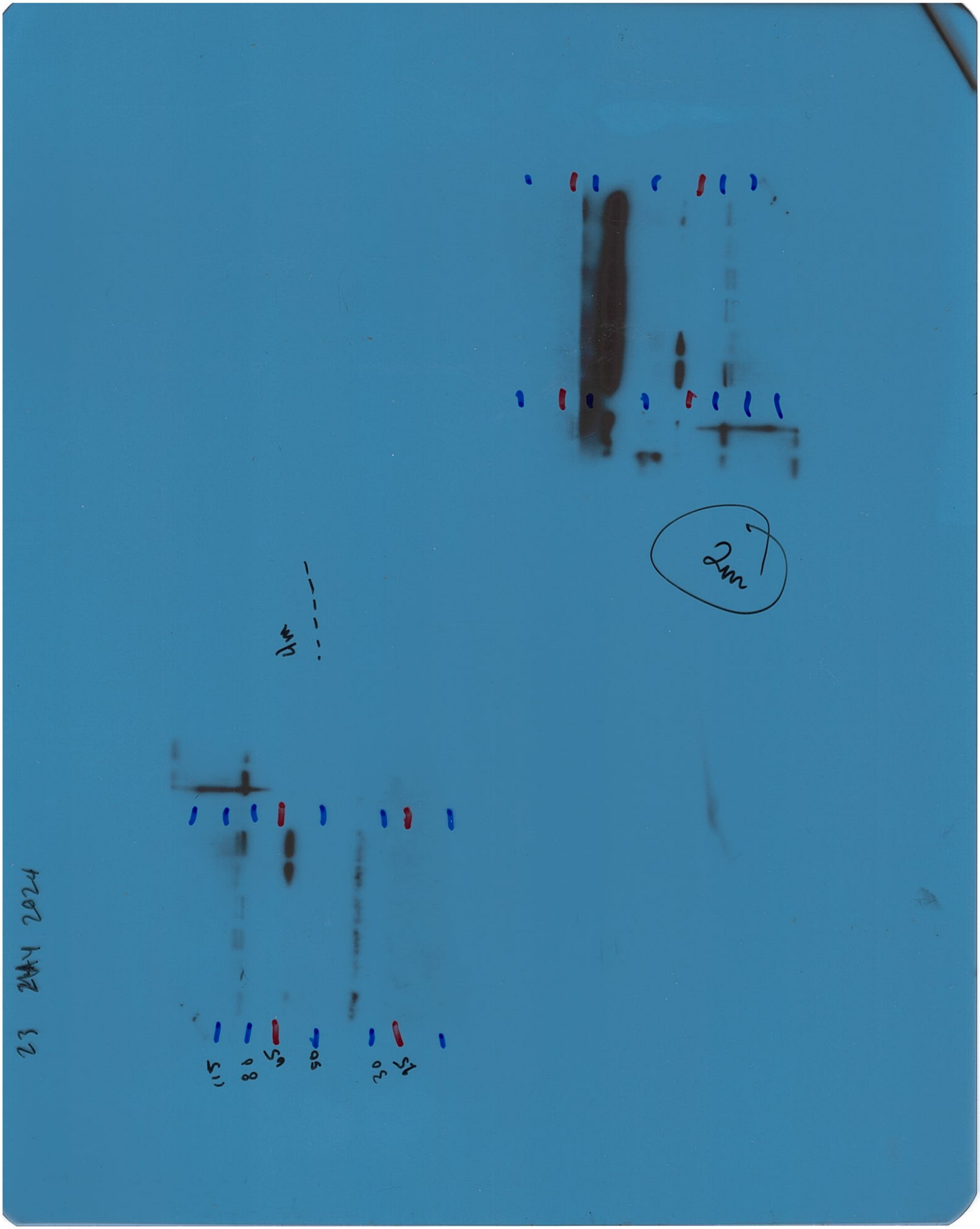
23 MAY 2024
115
80
65
50
30
25
4m
2m

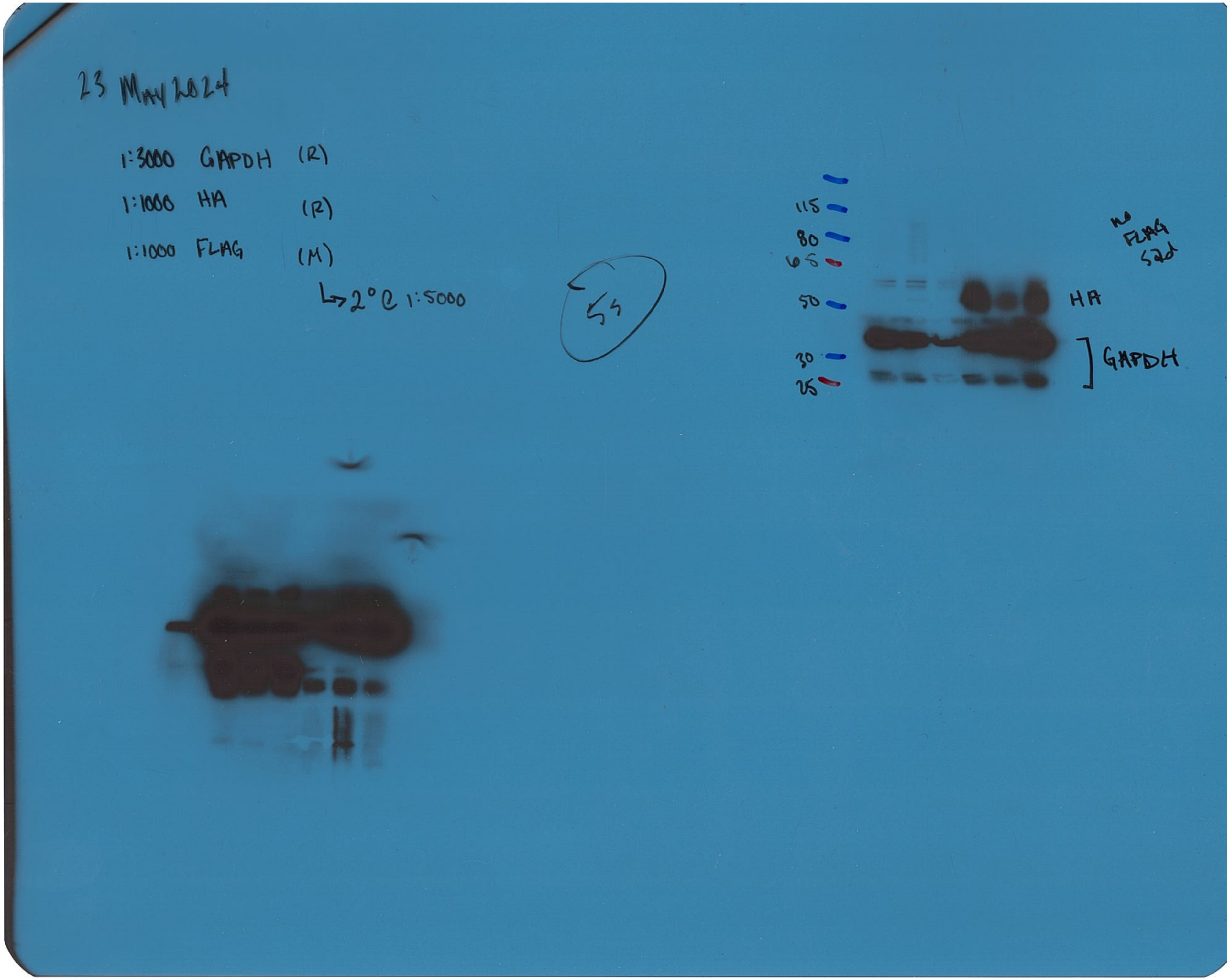
23 May 2024
1:3000 GAPDH (R)
1:1000 HA (R)
1:1000 FLAG (M)
↳ 2° @ 1:5000
55
115
80
65
50
30
25
HA
GAPDH
no FLAG 52d
