# Supplementary material for: The FAM53C/DYRK1A axis regulates the G1/S transition of the cell cycle
Source: eLife. 2026 Apr 30;14:RP109708. doi: 10.7554/eLife.109708 (PMC13132546; doi:10.7554/eLife.109708)
Supplement: Figure 4—figure supplement 1—source data 2. [file elife-109708-fig4-figsupp1-data2.zip › Figure 4 - figure supplement 1 - Source data 2/Xerox Scan_08052025135450 with marks.pdf]

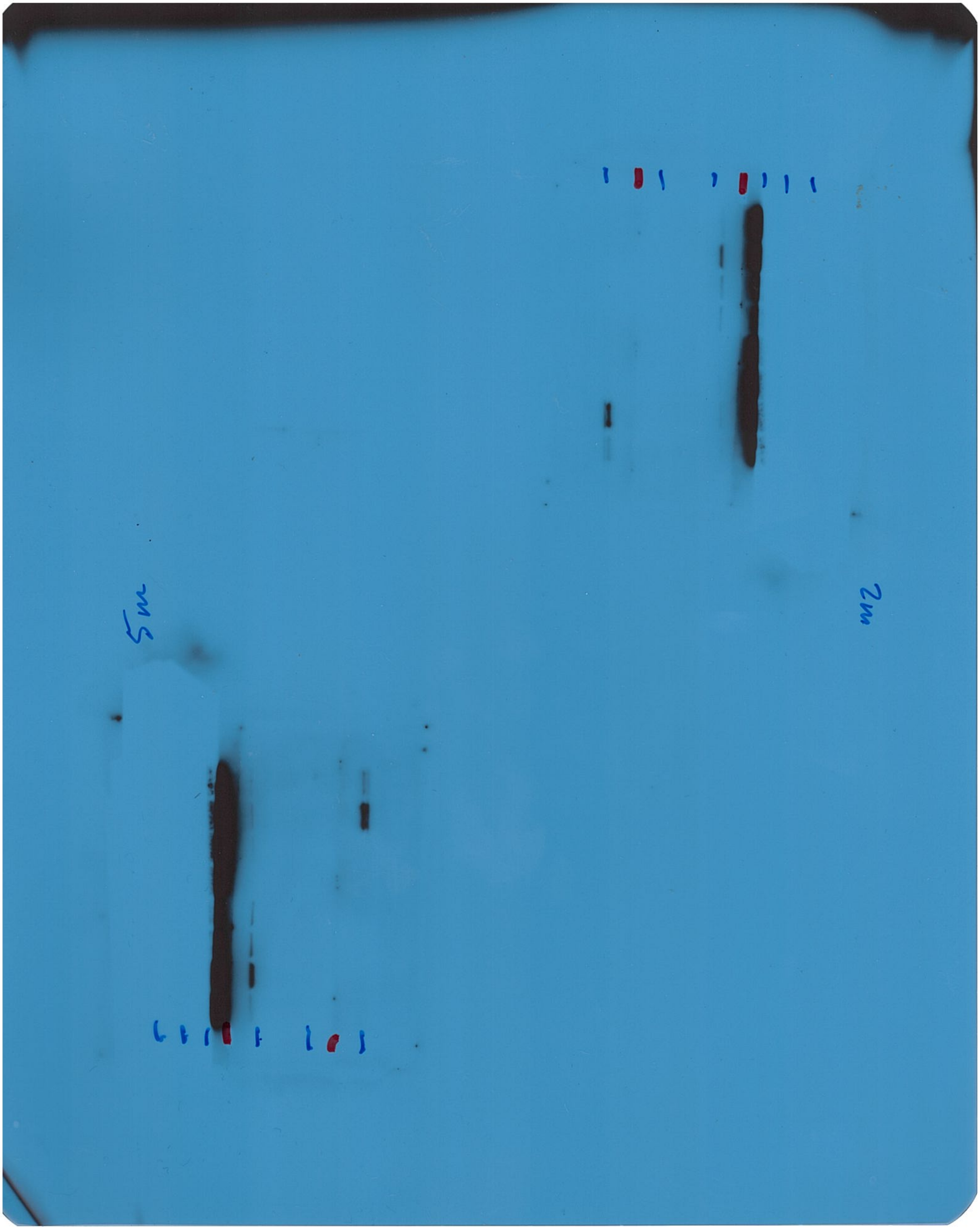
5m
2m

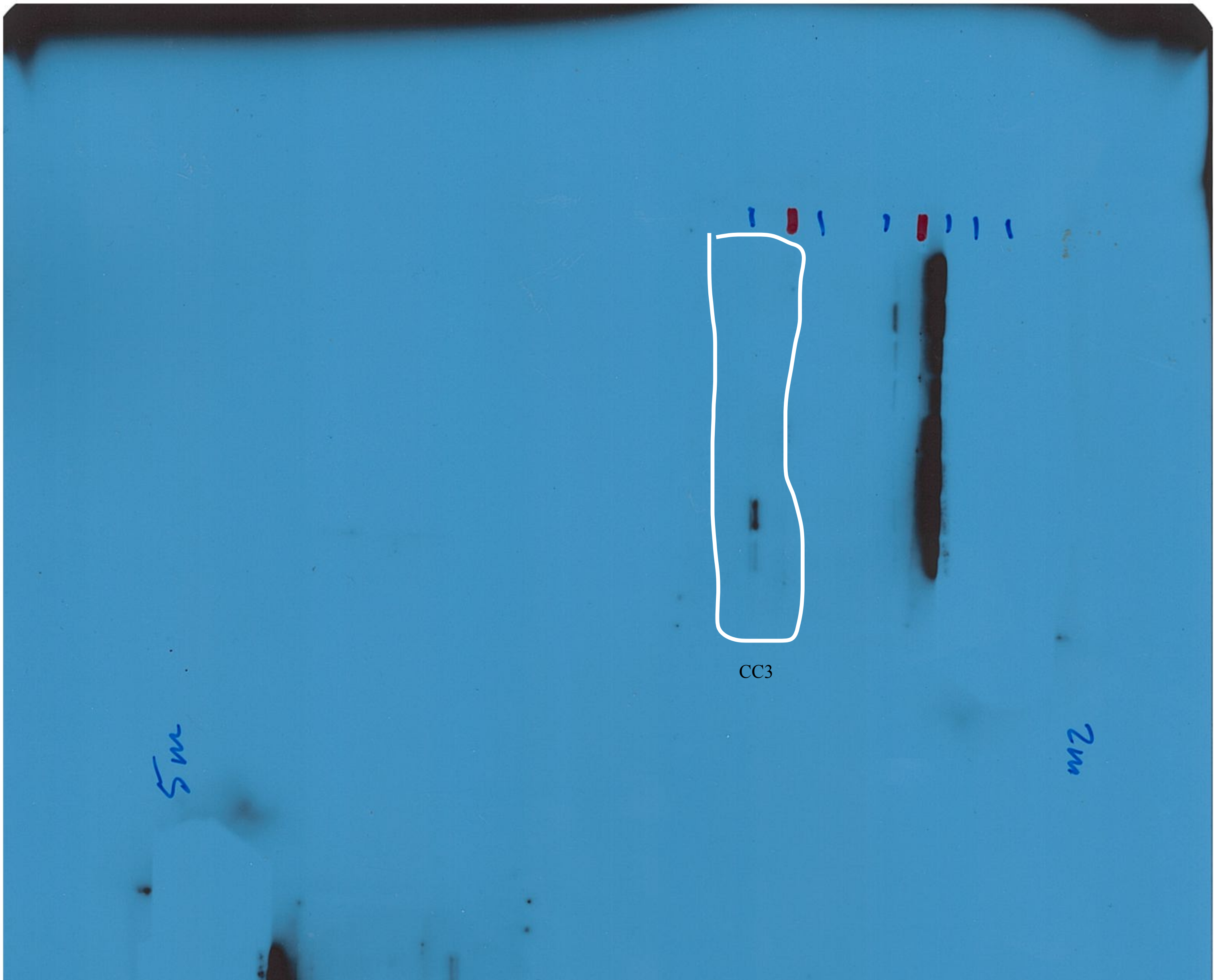
CC3

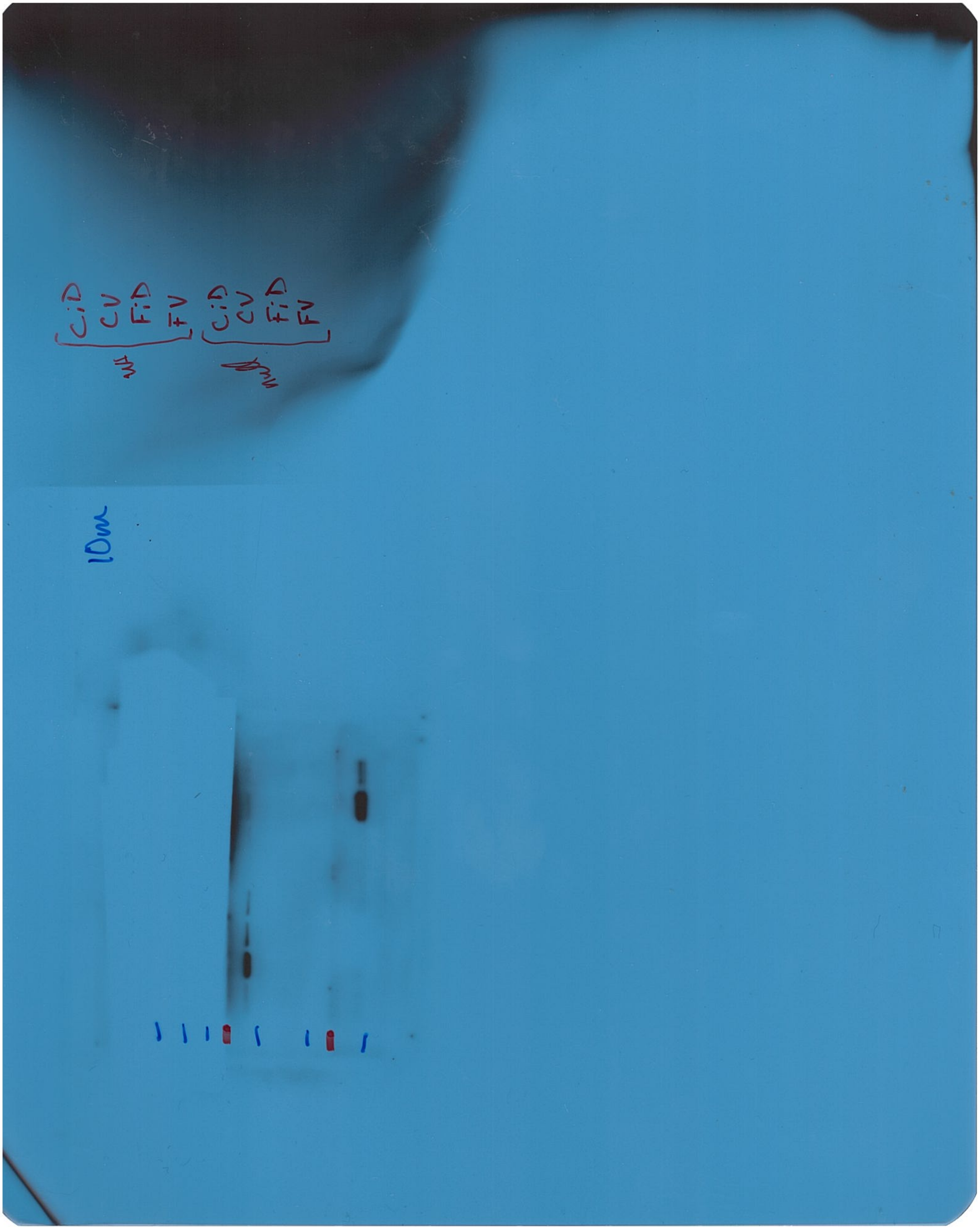
C:D
CV
F:D
FV
C:D
CV
F:D
FV

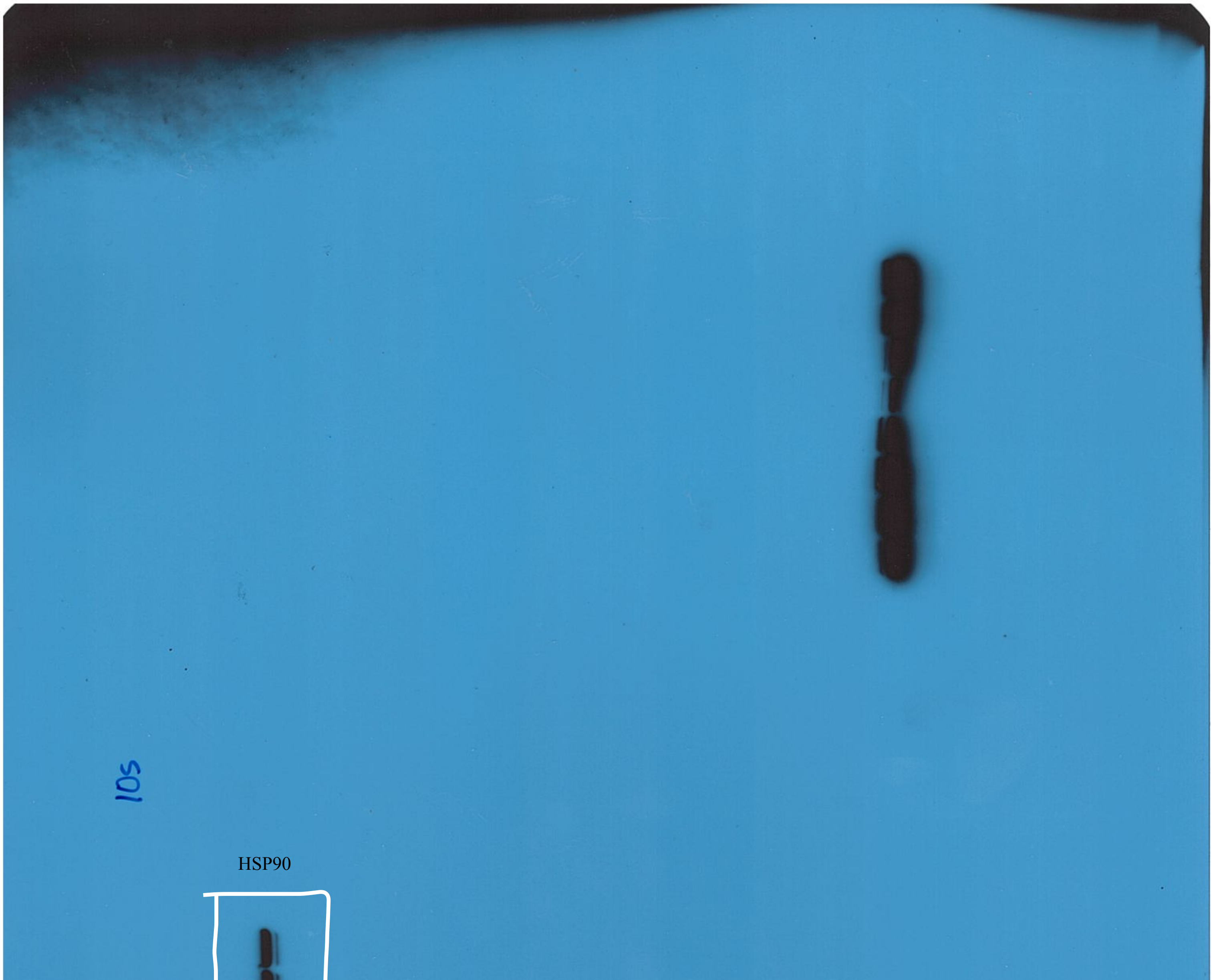
10s
HSP90
